# Clinical Hematochemical Parameters in Differential Diagnosis between Pediatric SARS-CoV-2 and Influenza Virus Infection: An Automated Machine Learning Approach

**DOI:** 10.3390/children10050761

**Published:** 2023-04-22

**Authors:** Dejan Dobrijević, Jelena Antić, Goran Rakić, Jasmina Katanić, Ljiljana Andrijević, Kristian Pastor

**Affiliations:** 1 Faculty of Medicine, University of Novi Sad, 21000 Novi Sad, Serbia; 2 Institute for Children and Youth Health Care of Vojvodina, 21000 Novi Sad, Serbia; 3 Faculty of Technology, University of Novi Sad, 21000 Novi Sad, Serbia

**Keywords:** COVID-19, SARS-CoV-2, influenza, diagnosis, laboratory parameters, machine learning

## Abstract

Background: The influenza virus and the novel beta coronavirus (SARS-CoV-2) have similar transmission characteristics, and it is very difficult to distinguish them clinically. With the development of information technologies, novel opportunities have arisen for the application of intelligent software systems in disease diagnosis and patient triage. Methods: A cross-sectional study was conducted on 268 infants: 133 infants with a SARS-CoV-2 infection and 135 infants with an influenza virus infection. In total, 10 hematochemical variables were used to construct an automated machine learning model. Results: An accuracy range from 53.8% to 60.7% was obtained by applying support vector machine, random forest, k-nearest neighbors, logistic regression, and neural network models. Alternatively, an automated model convincingly outperformed other models with an accuracy of 98.4%. The proposed automated algorithm recommended a random tree model, a randomization-based ensemble method, as the most appropriate for the given dataset. Conclusions: The application of automated machine learning in clinical practice can contribute to more objective, accurate, and rapid diagnosis of SARS-CoV-2 and influenza virus infections in children.

## 1. Introduction

Since December 2019, severe acute respiratory syndrome coronavirus-2 (SARS-CoV-2) has been causing an ongoing global pandemic. During this period, various pharmacological and non-pharmacological measures have been applied to suppress the virus. Among the non-pharmacological measures, the most important measure was the use of facial masks to help prevent the spread of the virus.

Increased public health surveillance, including social distancing, led to the significant suppression of other respiratory infections [1,2]. Today, we witness the relative relaxation of the aforementioned epidemiological measures. Consequently, children are more likely to fall ill with influenza A and B viruses and have more severe clinical symptoms, and the need for their hospitalization is more frequent. This phenomenon has been labeled “epidemiological debt” [3,4].

The influenza virus and SARS-CoV-2 have similar transmission characteristics: direct human-to-human contact via airborne droplets. Moreover, both infections present the same initial clinical picture: fever, malaise, cough, rhinitis, headache, etc. For these reasons, it is very difficult to distinguish them clinically [5]. The gold standard in virological diagnostics is the molecular detection of the viral genome via a polymerase chain reaction (PCR) test. However, the disease caused by a new strain of beta coronavirus (COVID-19) has significantly reduced the capacity of the health systems. The time required to obtain results is often prolonged, especially in developing countries [6]. Laboratory and clinical staff are overburdened, and the financial system is notably affected. Healthcare workers are faced with difficulty of quickly triaging patients until the first molecular diagnostics results arrive [7].

Due to the need for fast and, above all, affordable diagnostics during the COVID-19 pandemic, a renewed interest in machine learning algorithms within the frame of healthcare (systems) occurred [8]. Machine learning is the systematic study of algorithms and systems that improve their knowledge or performance with experience. Multidisciplinary in nature, machine learning draws on concepts and results from various fields, some of which include statistics, artificial intelligence, information theory, biology, cognitive science, and optimization theory. The impact of applying these approaches in medicine is so strong that the question arises whether computers will one day be able to replace staff [8,9].

The aim of this study was to determine if baseline hematochemical parameters could aid in achieving a differential diagnosis between SARS-CoV-2 and influenza virus infection in infants via an automated machine learning approach.

## 2. Materials and Methods

The study was conducted at the Institute for Children and Youth Health Care of Vojvodina, Novi Sad, Serbia. This cross-sectional study included a total of 268 infants treated for respiratory diseases in the period from June 2022 to January 2023. Of the infants, 133 were diagnosed with SARS-CoV-2 infection and 135 were diagnosed with influenza virus infection. Detection of the SARS-CoV-2 virus was performed via PCR technique, while the lateral immunochromatography method was used to detect the influenza virus antigen (CerTest, Biotec, Zaragoza, Spain). The criteria for exclusion from the study included: missing data, chronic and hematological diseases, and malignancy.

### 2.1. Data Acquisition

Data on baseline hematochemical parameters were collected on the day of admission: a complete blood count with differential, AST, ALT, LDH, and CRP. The samples for hematological analyses were collected using violet-topped evacuated tubes (0.5 mL) with ethylene-ediaminetetraacetic acid dipotassium salt dehydrate (K_2_EDTA) as a blood-clotting inhibitor (Becton Dickinson, Franklin Lakes, NJ, USA), while the samples for biochemical analyses were collected using yellow-topped evacuated tubes (0.5 mL) with clot activator (Becton Dickinson, Franklin Lakes, NJ, USA). The values were tested on the XN-1000 hematology analyzer (Sysmex, Kobe, Hyogo, Japan) and the DxC 700 AU chemistry analyzer (Beckman Coulter, Brea, CA, USA).

### 2.2. Baseline Statistical Analyses

Descriptive and inferential statistical methods were applied for data processing using the Statistical Package for the Social Sciences (SPSS) software (version 26.0; IBM Corporation, Armonk, New York, NY, USA). For discrete and continuous variables, distribution normality was estimated by the Shapiro–Wilk test. Between-group differences were analyzed using the independent samples t-test and Mann–Whitney U test. The significance level was set at 0.05.

### 2.3. Machine Learning Algorithms

As a part of data preprocessing, all outliers were identified by graphing the dataset (asterisks in boxplots) and removed from further analysis. Second, Spearman’s correlation was used to identify highly correlated variables. Parameters with correlation coefficients whose magnitudes were between 0.4 and 1.0 were excluded. A correlation heatmap was used to visualize the strength of relationships between the parameters (Figure S2). Third, min-max normalization was performed in order to put all scaled data in the range (0, 1). Thereafter, the Waikato Environment for Knowledge Analysis (WEKA) open-source software (version 3.8.6; University of Waikato, Hamilton, New Zealand) [10] was used to create the following machine learning models: support vector machine, random forest, k-nearest neighbors, logistic regression, neural network, and an automated machine learning model. The total number of instances, i.e., the reduced number of attributes in this model, was set to 10. The target attributes were SARS-CoV-2 and influenza. A 10-fold cross-validation was used to check the accuracy of the model. The performance of each algorithm was evaluated by calculating the accuracy, sensitivity, specificity, positive predictive value, and negative predictive value from the confusion matrix, i.e., a table of predicted and actual values of a classifier. An automated algorithm was created employing a state-of-the-art Bayesian optimization method, thereby solving the combined algorithm selection and hyperparameter optimization (CASH) problem, with a memory limit of 1024 MB, batch size 100, and 123 seeds.

### 2.4. Ethical Approval Statement

The study was approved by the Ethics Committee of the Institute for Child and Youth Healthcare of Vojvodina (22 July 2022; No. 3280-2).

## 3. Results

Based on the exclusion criteria, 268 infants were included in the study (Table 1). All infants were treated at the Institute for Children and Youth Health Care of Vojvodina, Novi Sad, Serbia, in the period from June 2022 to January 2023. The first group (COVID group, *n* = 133) consisted of infants with a SARS-CoV-2 infection. The median age of the group was 3.6 months, with a female share of 39.4%. The second group (influenza group, *n* = 135) consisted of infants with an influenza A or B infection. The median age of the group was 4 months, with a female share of 54.3%.

### 3.1. Clinical Laboratory Features

After excluding laboratory parameters with a Spearman’s rank correlation coefficient over 0.4, a total of 10 variables were included in the analysis: WBC, RBC, MCHC, RDW, PLT, MPV, EOS#, AST, LDH, and CRP (Figure S1). Statistically significant differences, determined using baseline statistical analysis, were observed only for the WBC parameter (*p* = 0.024). Infants with an influenza virus infection had higher values (12 ± 4.8 × 10^9^/L) than infants with a SARS-CoV-2 infection (9.1 ± 5.1 × 10^9^/L). Since a single parameter is not specific enough to distinguish between SARS-CoV-2 and influenza virus infections in infants, a set of 10 parameters were further employed to create and compare various machine learning models. These were able to reveal hidden relationships that existed among complex datasets using a multivariate/multiparameter approach.

### 3.2. Machine Learning Algorithm Performances

A comparison of six machine learning algorithms was carried out based on the standard evaluation metrics: sensitivity, specificity, positive predictive value, and negative predictive value (Table 2). An automated model convincingly outperformed other models, with an accuracy of 98.4%. The support vector machine, random forest, k-nearest neighbors, logistic regression, and neural network models demonstrated an accuracy ranging from 53.8% to 60.7%. Furthermore, a comparison of the machine learning algorithms based on their F1-score, an additional measure of a test’s accuracy in the statistical analysis of binary classification, was performed (Figure S2). The automated machine learning model achieved the highest F1-score of 98.4%. Using a 10-fold cross-validation to evaluate the performance gains, the automated algorithm proposed a random tree model, i.e., a randomization-based ensemble method, as the most suitable classification model for the given dataset.

## 4. Discussion

Th early recognition of SARS-CoV-2 infection remains a diagnostic challenge, especially in pediatrics. In order to begin treatment and prevent the spread of infection, it is necessary to identify positive patients as quickly as possible [11,12]. With the development of information technologies, the possibilities for the application of intelligent software systems in disease diagnosis and patient triage increase [8,9].

In this study, an automatic machine learning algorithm was applied. This type of data processing is particularly suitable for researchers who are not data scientists and want to use machine learning methods in their research. Automatic machine learning enables the automatic selection of the best algorithm and its automatic optimization based on hyperparameter settings. Specifically, Auto-WEKA considers various learning algorithms Ά = {A(1), ..., A(k)} and their spaces of hyperparameters Ʌ(1), ..., Ʌ(k), aiming to find the best combination of algorithm A(j) ϵ A and hyperparameters λ ϵ Ʌ (j) that will minimize cross-validation loss:

(1) $$A\;\in \;\begin{array}{c} \mathrm{argmin}\\ A\;\in \;Ά \end{array}\;\frac{1}{k}\;\overset{k}{\underset{i=1}{\sum }}L\left(A,D_{\mathrm{train}}^{\left(i\right)},D_{\mathrm{test}}^{\left(i\right)}\right)$$

where $L\left(A,D_{\mathrm{train}}^{\left(i\right)},D_{\mathrm{test}}^{\left(i\right)}\right)$ represents the cross-validation loss achieved by algorithm A with hyperparameters λ, which are trained on $\;\left(D_{\mathrm{train}}^{\left(i\right)}\right)$ and furhther evaluated on $\left(D_{\mathrm{test}}^{\left(i\right)}\right)$ [13,14,15].

The use of machine learning processing on routine laboratory tests as input datasets was the main focus of many research studies during the COVID-19 pandemic [16,17,18,19,20,21,22,23]. For example, Caires Silveira E. [16] employed the XGBoost classifier to develop a machine learning model for the prediction of COVID-19 from hemogram results. The predictive model obtained an accuracy of 80%, with a sensitivity of 75.6% and a specificity of 82%. The variables with the greatest influence on the predictive decision were BASO#, EOS#, and WBC. Kukar et al. [17] used a routine blood test results dataset of 160 SARS-CoV-2 positive patients and 5333 control patients with other non-COVID-19 infections to develop a machine learning model, thereby achieving a sensitivity of 81.9% and a specificity of 97.9%. In a study conducted by Babaei Rikan et al. [23], routine laboratory blood tests were used to construct a deep neural network model as a supplementary tool for diagnosing COVID-19 with an accuracy of 92.11%.

In the proposed study, the following data from the laboratory findings of patients were used as input variables: a complete blood count with differential, AST, ALT, LDH, and CRP. After screening out highly correlated variables (Spearman’s rank correlation coefficient over 0.4), a sum of 10 variables was obtained: WBC, RBC, MCHC, RDW, PLT, MPV, EOS#, AST, LDH, and CRP, and included in further data processing. While common machine learning algorithms, such as the support vector machine, random forest, k-nearest neighbors, logistic regression, and neural network algorithms, achieved an accuracy between 53.8% and 60.7%, the automated machine learning tool applied in this study constructed a model that reached an accuracy of 98.4%.

Traditional machine learning methods are challenging and time-consuming. Automated machine learning makes it easy to build and use machine learning models in the real world by automatically testing and selecting the best algorithm on raw data. This is why researchers currently embrace this approach in data curation [24,25,26]. For example, a study by Ikemura et al. [24] aimed to use an automated machine learning approach to train various demographic and laboratory variables in order to predict mortality in COVID-19 patients. Tran et al. [25] evaluated the analytical performance of the MALDI-TOF (matrix assisted laser desorption/ionization-time of flight) mass spectrometry method for screening COVID-19 patients in which output data were further analyzed by an automated machine learning approach. In a study conducted by Papoutsoglou et al. [26], an automated machine learning approach was employed to analyze proteomic, metabolomic, and transcriptomic measurements in order to discriminate severe from non-severe COVID-19 patients and to identify COVID-19 patients from both those having another acute respiratory condition and virus-free individuals.

Automated machine learning is a powerful tool which can help medical professionals to detect diseases, i.e., COVID-19, at an early stage. It has the potential to provide a more accurate result. Additionally, it saves time and money, which is important for facility management and, most importantly, for saving lives.

Supported by the above-mentioned authors’ evidence and similar clinical laboratory studies, it is safe to assume that automated machine learning is an effective and informative tool for generating preliminary clinical decisions regarding COVID-19.

However, there are certain limitations of the proposed approach and some practical considerations to be considered for future research. First, this was a single-center study. Therefore, only a limited number of children could be included. Second, all pediatric patients in this study belonged to a European population. Additional multinational and multiethnic studies could provide insight into the performance of the developed model on a global level. Third, all children with underlying conditions were excluded from this study, making its clinical applicability for children with coinfections limited.

## 5. Conclusions

Machine learning is shown to be a powerful tool that plays a significant role in medicine and medical diagnostics. Although the solutions are still not at a high enough level to be able to replace expert assessment, their application in clinical practice can contribute to a better, more objective, and efficient diagnosis of SARS-CoV-2 and influenza virus infections in children.

## Figures and Tables

**Table 1:** Demographic and laboratory data within groups.

Variable	COVID Group (*n* = 133)	Influenza Group (*n* = 135)	Overall (*n* = 268)	*p*-Value
Female/Male (n) ^a^	63/70	69/66	132/136	0.094
Age (m) ^b^	3.6 (2.15–5.4)	4 (2.6–7.8)	3.7 (2.2–6.4)	0.298
WBC (10^9^) ^c^	9.1 ± 5.1	12 ± 4.8	10.5 ± 5.1	**0.024**
RBC (10^12^) ^c^	3.8 ± 0.6	4.1 ± 0.5	3.9 ± 0.6	0.058
MCHC (g/L) ^c^	344 ± 18	344 ± 12	344 ± 15	0.871
RDW (%) ^b^	11.8 (10.9–13.2)	11.8 (10.5–12.7)	11.8 (10.7–12.9)	0.333
PLT (10^9^) ^b^	411 (340–523)	366 (331–448)	384 (331–492)	0.222
MPV (fL) ^c^	7.7 ± 1	7.4 ± 0.9	7.5 ± 1	0.296
EOS# (10^9^) ^b^	0.09 (0.06–0.13)	0.14 (0.08–0.3)	0.1 (0.06–0.23)	0.06
AST (µkat/L) ^b^	0.79 (0.7–0.95)	0.7 (0.61–0.85)	0.74 (0.67–0.91)	0.076
LDH (µkat/L) ^b^	4.49 (4.31–5.08)	5.02 (4.33–5.78)	4.73 (4.32–5.55)	0.163
CRP (mg/L) ^b^	2.2 (1–10.3)	2.2 (0.7–5.8)	2.2 (0.7–8.5)	0.971

^a^ Values are numbers; Chi-square test. ^b^ Values are median (interquartile range: Q1–Q3); Mann–Whitney U test. ^c^ Values are mean ± standard deviation; independent samples *t*-test. WBC—white blood cells; RBC—red blood cells; MCHC—mean corpuscular hemoglobin concentration; RDW—red blood cells distribution width; PLT—platelet; MPV—mean platelet volume; EOS#—absolute eosinophil count; AST—aspartate aminotransferase; LDH—lactate dehydrogenase; CRP—C-reactive protein; Value in bold is statistically significant.

**Table 2:** Machine learning classifiers for differential diagnosis between pediatric SARS-CoV-2 and influenza virus infection.

Classifier	Accuracy (%)	Sensitivity (%)	Specificity (%)	Positive Predictive Value (%)	Negative Predictive Value (%)
Automated machine learning	98.4	96.9	100	100	96.7
Support vector machine	60.7	59.4	62.1	61.0	60.4
Random forest	59.0	59.4	58.6	58.9	59.1
k-nearest neighbors	55.9	53.1	58.6	56.2	55.6
Logistic regression	53.8	59.4	48.3	53.4	54.3
Neural network	53.8	59.4	48.3	53.4	54.3

## Data Availability

Research data are not shared.

## Supplementary Materials

The following supporting information can be downloaded at: https://www.mdpi.com/article/10.3390/children10050761/s1, Figure S1: Correlation heatmap. Figure S2: Comparison of different machine learning algorithms based on their F1-score.
