# Supplementary figures and images for: Clinical Hematochemical Parameters in Differential Diagnosis between Pediatric SARS-CoV-2 and Influenza Virus Infection: An Automated Machine Learning Approach
Source: Children (Basel). 2023 Apr 22;10(5):761. doi: 10.3390/children10050761 (PMC10217039; doi:10.3390/children10050761)

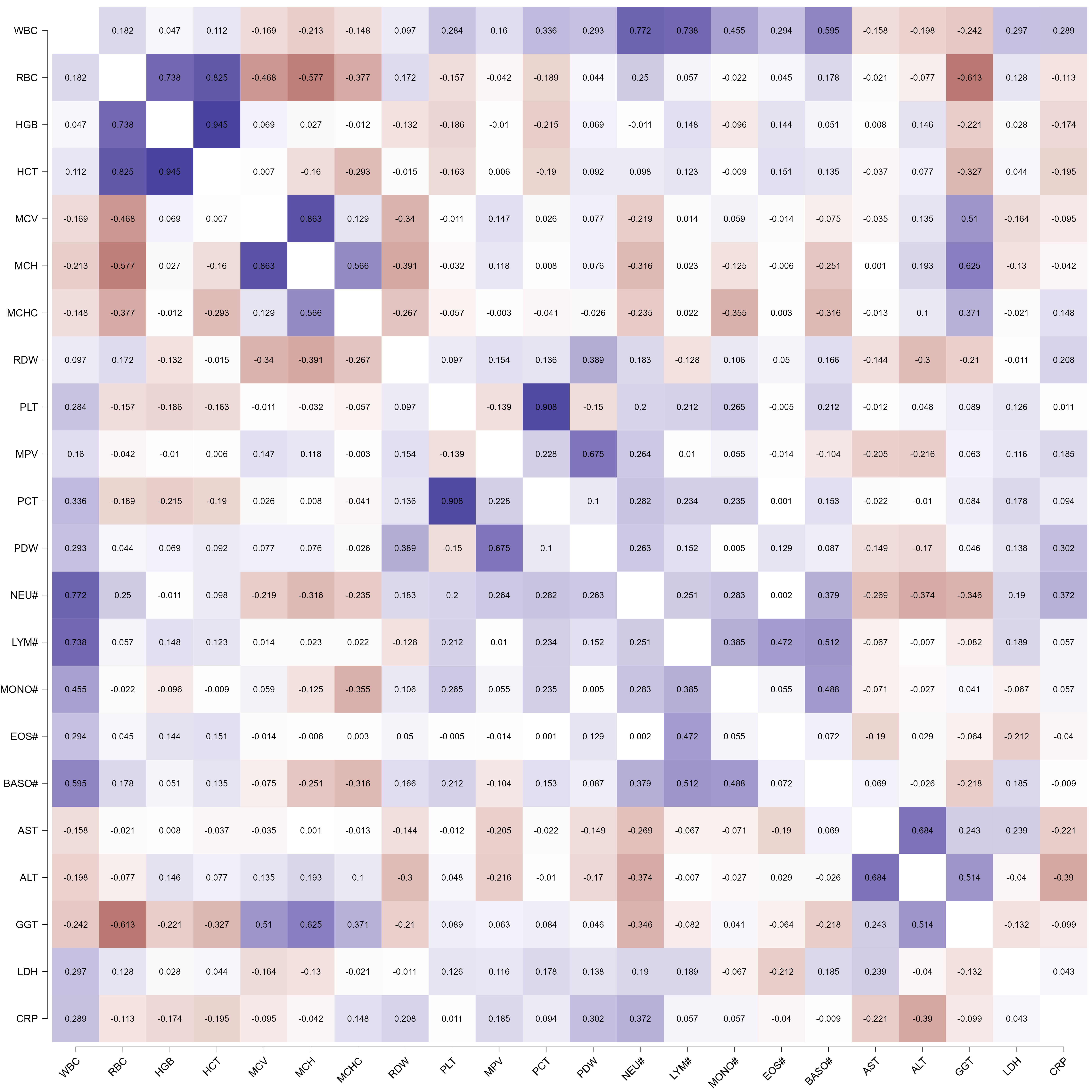

Supplement: Supplementary file 1 [file children-10-00761-s001.zip › Figure S1. Heatmap.png]

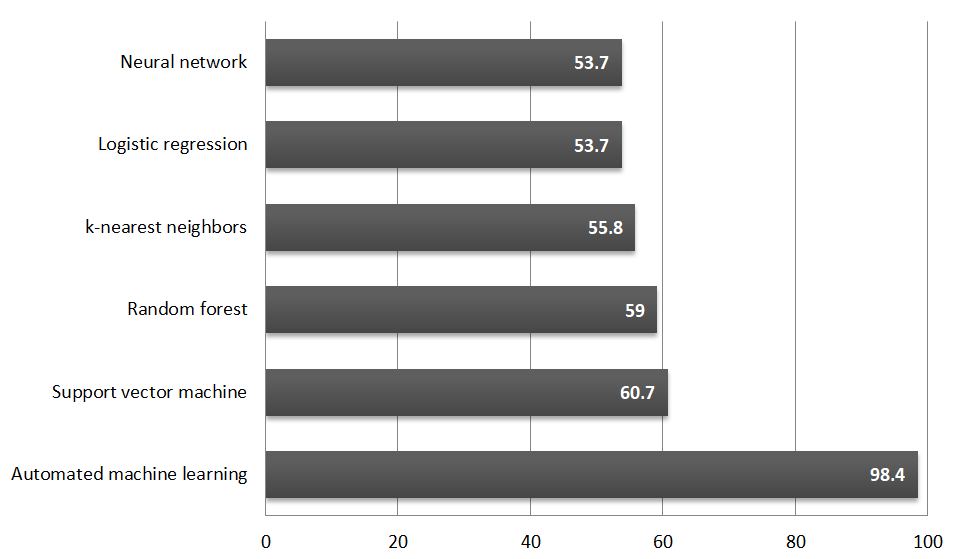

Supplement: Supplementary file 1 [file children-10-00761-s001.zip › Figure S2. F1-scores.png]
